# Conservatory Musicians’ Temporal Organization and Self-Regulation Processes in Preparing for a Music Exam

**DOI:** 10.3389/fpsyg.2020.00089

**Published:** 2020-02-03

**Authors:** Roberta Antonini Philippe, Céline Kosirnik, Noémi Vuichoud, Terry Clark, Aaron Williamon, Gary E. McPherson

**Affiliations:** ^1^PHASE Lab, Institute of Sport Sciences, University of Lausanne, Lausanne, Switzerland; ^2^Centre for Performance Science, Royal College of Music, London, United Kingdom; ^3^Melbourne Conservatorium of Music, The University of Melbourne, Melbourne, VIC, Australia

**Keywords:** self-regulation learning, organization, music students, performance exam, Australia

## Abstract

Performing at the very highest levels requires rigorous preparation before the important performance. Musicians and especially music students encounter many challenges when preparing themselves for an important musical performance. This study sought to identify and analyze the context-specific temporal organization and self-regulation efforts that music students employ during their preparation period. Conservatory musicians were recruited from an Australian University Conservatorium. Thirteen conservatory musicians aged between 19 and 21 years (*M* = 19.6; SD = 0.76) participated in the study. All musicians, through an elicitation interview, were asked to recall and reconstruct their preparation period, leading up to a performance exam. Elicitation interviews provided access to music students’ experiences by describing their general preparation. The results showed that conservatory musicians go through different phases (Phase 1: Choosing a piece; Phase 2: Piece discovery; Phase 3: Piece interpretation; Phase 4: Performance preparation). Self-regulatory efforts to prepare for a music performance exam vary from one musician to another. Organizational and disorganizational competencies, specific self-regulatory skills, seem not to be exploited by conservatory musicians. Also, during their preparation, most music students prefer technical and musical work than challenges such as playing in front of the public. Emotionally, conservatory musicians go through pleasant and unpleasant emotions depending on the phase of their preparation. Our results show that music students could benefit from advice on how to organize their preparation period well before an important performance takes place. Implications for conservatory musicians and teachers are discussed.

## Introduction

Elite musicians need to overcome many challenges and invest much time practicing in order to excel and perform at their highest level, especially during their formative years. Deliberate practice, which was first studied in sport sciences, is also important in the music domain (Ericsson et al., 1993; Lehmann and Ericsson, 1997; Miksza and Tan, 2015). However, the deliberate practice framework, which has been simplified within non-research literature as the 10-year and 10,000-h rule (Kaufman, 2013), is becoming increasingly contested. Some evidence suggests that it explains no more than 30% in the expertise of a performer (Meinz and Hambrick, 2011; Hambrick et al., 2013; Macnamara et al., 2014). Indeed, the quality rather than quantity of practice is regarded by some as more important in explaining achievement (Ryan and Deci, 2000, 2002; McPherson et al., 2016). Research has also found that successful performances are often connected with feelings of sufficient preparation, positive mindsets and presenting a high, yet attainable, level of challenge. In contrast, less successful performances appear to be linked with inadequate preparation, negative mental outlooks, frustration, and a lack of enjoyment during the performance itself (Clark et al., 2014). Therefore, it is important to investigate the performance preparation phase that impacts on the performance itself, especially given that research on practice and learning within conservatory musicians is still limited compared to sport sciences (Nielsen, 2001; Miksza, 2015; McPherson et al., 2019), another field in which public performance is a key point.

In addition, research often focuses on the remediation of performance anxiety (Rae and McCambridge, 2004; Kenny, 2011; Studer et al., 2011; Kenny and Ackermann, 2015) and less on how music performers employ strategies directed at optimizing desired mental and emotional experiences during the preparation performance in order to develop their talent. In sport sciences, talent development is widely studied (MacNamara et al., 2006, 2008; MacNamara et al., 2010; Collins et al., 2016; Johnston et al., 2018). Characteristics and specific competencies have been outlined to develop elite level athletic attributes: goal setting, coping with pressure, planning and organization skills, the quality of practice and realistic performance evaluations (Pecen et al., 2016, 2018). However, a recent study showed that musicians lack different competencies and highlighted the importance of being able to organize preparation for a performance (Pecen et al., 2018).

Researchers have also been interested in the general steps and learning skills musicians require to develop their ability to play a piece “perfectly” (Reid, 2005). Many activities are required to learn a piece: repetition, memorization, the development of a technical expertise, and ultimately the formulation of interpretation (Rink and Rink, 2002; Reid, 2005; Lehmann and Jørgensen, 2012; Jørgensen and Hallam, 2016). Learning a piece appears to be different from one musician to another, because they apply different activities designed to facilitate learning or to improve music performance in specific areas (Barry and Hallam, 2002; Lehmann et al., 2007).

More than 60 years ago, researchers tried to identify key phases when learning a piece (Wicinski, 1950; Fitts and Posner, 1967). The steps identified by these researchers to learn a piece varied and are dealt with differently depending on each musician, but what emerges overall is that musicians apparently go through general learning phases that are interrelated to each other (Antonini Philippe and Güsewell, 2016). The first step is to choose pieces and work on them. Pieces can be imposed, selected in a list of possible pieces, or freely chosen. The second step involves the technical work required to master the piece. At this stage, the piece is worked through in sections, the time spent on a piece depends on its complexity and this often increases gradually as the practice progresses. Memorization at this stage goes through a structuring process, usually as an internal mapping of the work (Williamon, 2002). Musicians must develop a flexible memory retrieval system that will permit the performance to continue, whatever may go wrong (Ginsborg et al., 2006; Noice et al., 2008). In the third step, the repertoire is worked on as a whole, and musicians work on refining the interpretative details and overcoming technical problems. The fourth step is partly a maintenance procedure, which involves subtle modifications in interpretation, memory, or technique (Lehmann et al., 2007). Some musicians plan interpretation at the outset, based on a study of the score or from ideas gleaned from listening to a wide range of music and different interpretations of the same piece (Hallam, 1995; Lisboa et al., 2005), primarily letting the musical and expressive idea guide the technical work (Sloboda, 2000; Chaffin et al., 2003). Other musicians develop a performance plan after mastering most of the technical challenges (Nielsen, 2001).

Work routines and a specific, dedicated amount of time allocated each day preparing for a competition are conducive to good learning (Jørgensen, 2004). However, these hours of practice need to be quality hours as mentioned by McPherson et al. (2016). Musicians also have to be able to evaluate their own musical performance in order to develop daily routines allowing them to set learning objectives, based on clear evaluation, in order to manage a public examination, concert, or recital (McPherson and Schubert, 2004; Antonini Philippe, 2013). No specific method can be recommended, but some points seem important for musicians to perform at their best: an adapted working technique (Jørgensen, 2004) and receiving feedback from a competent and empathetic person (Woody et al., 2018).

According to Panadero (2017), self-regulated learning (SRL) is a comprehensive and holistic conceptual framework that defines the cognitive, metacognitive, behavioral, motivational, and emotional/affective aspects of learning. Self-regulated learning theory also considers other variables, in particular self-efficacy, volition, and cognitive strategies that influence learning. Importantly, SRL involves cyclical and multi-layered processes comprising three complementary phases: planning, doing, and reflecting (Zimmerman, 1998; Upitis et al., 2010; McPherson and Zimmerman, 2011). Researchers interested in preparation and learning processes within conservatory musicians have pointed out the importance of self-regulation (McPherson et al., 2017, 2019). Previous studies have investigated how students acquire the tools necessary to take control of their own learning and thereby learn effectively (McPherson et al., 2019) or how they negotiate effectively the learning phases that continuously interact: forecasting (goals and beliefs about oneself), performance, and self-correction (Zimmerman, 2000; McPherson and Renwick, 2011). Participants who are persistent and reflect on their practice display more effective practice, and experience higher levels of flow that are associated with self-regulation (Williamon and Valentine, 2000). Self-regulation instructions often comprise: concentration, goal-selection, planning, self-evaluation, and rest/reflective activity (Miksza, 2015).

A key aspect of self-regulation is emotional management. Some studies have attempted to describe musicians’ thoughts and perceptions in successful and less successful performances (Pekrun et al., 2002; Clark et al., 2014). Emotions and other performance-related affective states are considered to arise from person-environment transactions, which comprise individuals’ goals, behaviors, attitudes, and motivations in that situation (Lazarus, 2000; Efklides, 2011; Efklides et al., 2018). When studying these phenomena, the self-regulation efforts that individuals use to alter their interaction with the environment in order to better meet their goals also need to be considered (Lazarus and Folkman, 1984; Lazarus, 2000). Therefore, in order to be well prepared for an evaluated performance, emotions should also be self-regulated during the preparation and learning process (Thomson and Jaque, 2017).

To date, research in music psychology has been conducted using large-scale surveys (McPherson and McCormick, 1999, 2000; Nielsen, 2004; Miksza, 2012; Araújo, 2016; Spahn et al., 2016), with context-specific and ecological methods being limited (Chaffin and Imreh, 1994, 1997, 2001; Nielsen, 2001, 2004; Chaffin et al., 2003; Chaffin and Logan, 2006; Leon-Guerrero, 2008; McPherson et al., 2019). More context-based research holds the potential to provide a better understanding of how musicians manage their progress toward their learning goals.

For this study, a specific approach to human activity was therefore used which emphasizes the different experiences “showable, narratable and commentable to an observer or interlocutor” meaningful to conservatory musicians, corresponding to “the activity of a particular [musician], engaged in a particular physical and social environment and belonging to a particular culture” (Theureau and Jeffroy, 1994, p. 19). In music contexts, a recent study investigated the simulation of orchestral competition using a phenomenological approach (Antonini Philippe and Güsewell, 2016), and attempted to be as close and as authentic as possible to these musicians’ experiences at each particular moment in the data collection period. Some researchers are starting to study musicians in a phenomenological way. Researchers are interested in being as close as possible to the individual’s reality, therefore approaches such as microanalysis through video (Cleary et al., 2012; McPherson et al., 2017) or elicitation interviews with traces (e.g., drawings, photos, etc.…) (Antonini Philippe and Güsewell, 2016) aim at better understanding what they were doing, thinking, or feeling in specific situations.

The aim of this study was to analyze the context-specific temporal organization and self-regulation efforts that music performers use during the preparation period leading up to an important performance. Self-regulation strategies that unfold in regard to performance cannot be understood without considering the specificity of the interaction between the situation and the individual (i.e., his actions). For these reasons, this research will use a phenomenological approach to examine how conservatory musicians prepare for an important music performance.

## Materials and Methods

### Participants

Thirteen young music students from an Australian University Conservatorium participated in this research, consisting of seven men and six women aged between 19 and 20 years (*M* = 19.6; SD = 0.76). These musicians played different instruments (flute = 3; clarinet = 2; piano = 4; guitar = 1; saxophone = 1 and voice = 2); however, all were undertaking the “classical music” stream of their undergraduate degree. Conservatory musicians were in their first to last year of enrolment in an undergraduate Bachelor of Music degree at the Conservatorium. Five musicians were in their Honors year, a supplementary year during which students can undertake highly specialized work in music performance, composition, jazz and improvisation, musicology, or ethnomusicology.

Participants were first asked about the total duration of their preparation process for a typical performance (e.g., recital, exam). Given that participants were undergraduate students studying music within a higher education institution, all performances that were discussed were those required as part of their studies within the Conservatorium.

### Procedure and Methods

A few months before the study, the music students were invited by email to participate in the research, on a voluntarily basis. Two types of data were collected to help build each musician’s experience database: (1) traces of past activity, using the graph drawn by conservatory musicians themselves and (2) recorded and transcribed data from the elicitation interviews.

**Figure 1:**
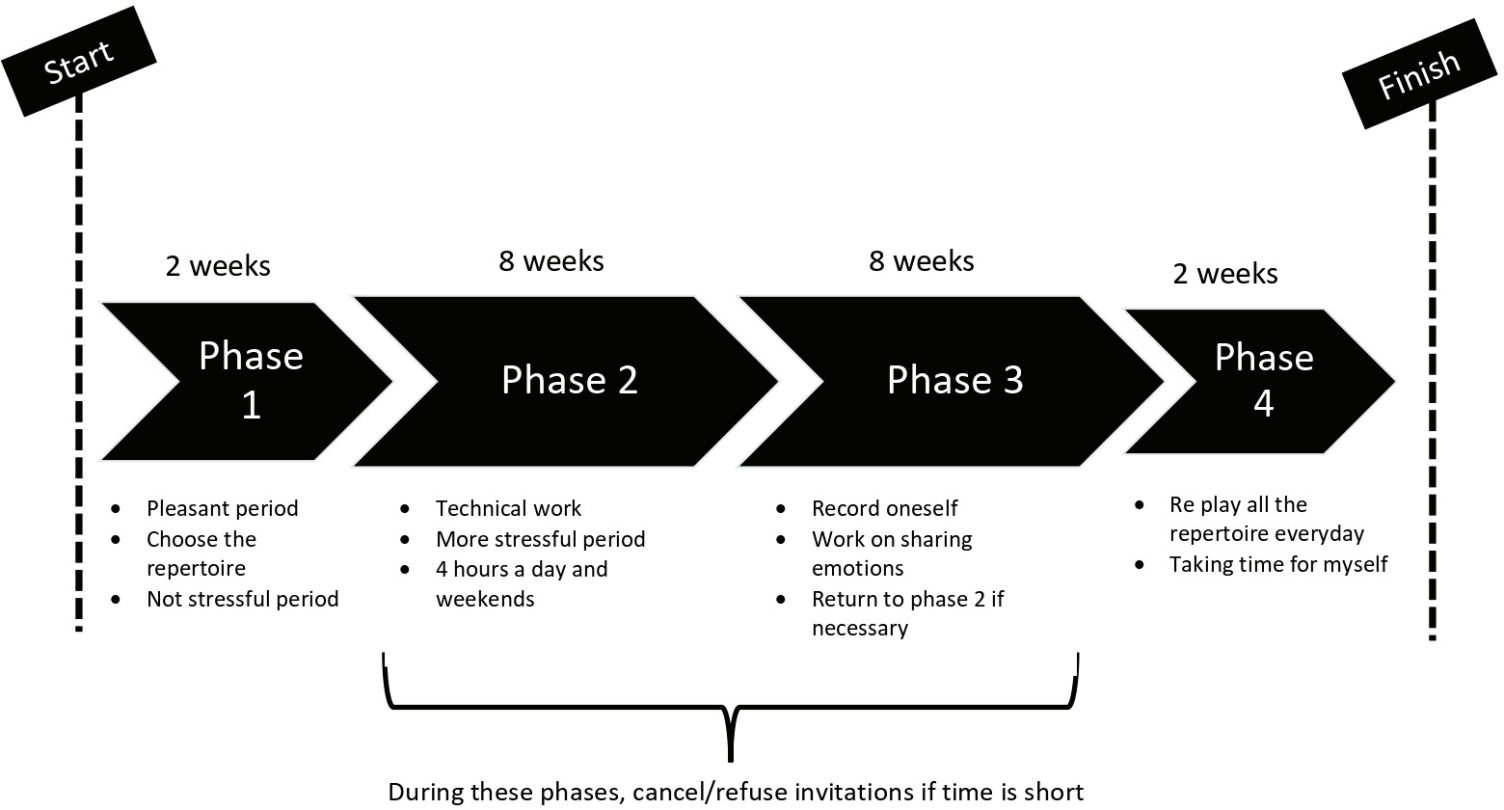
A reproduced graph, drawn by a musician (M1).

1. To recapture more precisely the temporal dynamics of the performance’s preparation, the participants were encouraged to represent their experience by drawing a graph (see Figure 1) and re-live the preparation states they experienced from the beginning of this performance until its end (Drasch and Matthes, 2013). The interviewer suggested illustrating the temporality of a preparation music performance and point out key phases within this preparation, by discussing their actions, emotions, and thoughts experienced. Each musician was encouraged to recall how they prepared for a performance. To collect experience data, *a posteriori*, elicitation interviews were used. This type of interview was tested in different sports studies that also dealt with the analysis of individual and collective experiences and their development over time (e.g., Antonini Philippe et al., 2016; Rochat et al., 2017). These methodological approaches were also used in the artistic field with the study of a contemporary musical composition (Donin and Theureau, 2008) and the study of the preparation for contests with musicians (Antonini Philippe and Güsewell, 2016).
2. The conservatory musicians participated in elicitation interviews to explore and identify the key steps they used to prepare for their performance. Elicitation interviews (Theureau, 2010) lasting between 60 and 120 min were used to elicit the conservatory musicians’ experiences. These interviews aimed at collecting verbal data on a situation previously experienced. This type of interview could also allow collection of gestural data; however, these data were not used for the study. During the interview, the conservatory musicians were encouraged to recall themselves again in this specific situation of a performance preparation, previously experienced, based on the graph they drew. The interviewer’s questions were about the preparation of the performance exam and its impact on the musicians, as for example: What do you do? How do you organize yourself to prepare the exam? What do you feel? What strategies do you put in place? How do you live this period of preparation? What are your concerns at that moment? What do you think about? These questions reflect behaviors, emotions, and cognitions; however, there was not any interview guide and the focus was on the temporality and general construction of the preparation.

The interviews were recorded digitally using an iPad. The audio recordings were then transcribed verbatim. Conservatory musicians participated voluntarily and could stop answering questions at any time if they felt uncomfortable. Written and informed consent was obtained from all participants attesting that the data could be analyzed and discussed for publishing. Also, this research project was approved by the Human Research Ethics Committee of The University of Melbourne. Verbatim and experiences collected through graphs were then analyzed to bring forth categories that correspond to the preparation of a performance exam. The first author conducted all interviews.

### Data Analyses

As the goal of this investigation was to establish a contextualized perspective of music students’ subjective experiences when preparing for a musical performance within the university context, the analysis procedure was data-driven rather than theory-driven (Charmaz, 2003). A thematic inductive approach was employed to analyze the data, which involved different steps (Braun and Clarke, 2006; Braun et al., 2016). The transcripts were first read through to familiarize the researchers with their content. Analysis involved identifying and dividing the transcripts into meaning units: parts of text representing a single idea in relation to the research question (Robson, 2011). These meaning units were labeled and then reviewed across all of the transcripts to check for consistency across the dataset. Next, the labeled meaning units were grouped into categories and themes with other similar meaning units. The themes were then grouped together into general dimensions that characterized the performance preparation process described by the participants. No software was employed in any stage of the analysis; instead all coding and grouping were conducted by hand.

Steps were taken to establish and ensure the reliability of the analysis process and the emergent findings. Discussion among the research team reviewed the resulting themes following the analysis process (Smith and McGannon, 2018). In the case of any disagreements, consolidation was sought through discussion and cross-referencing with the interview transcripts. To ensure that the researchers’ interpretation did not go beyond what was actually said by the participants, quotes are provided throughout the section “Results” below so that readers are able to form their own judgments on their meaning.

## Results

**Figure 2:**
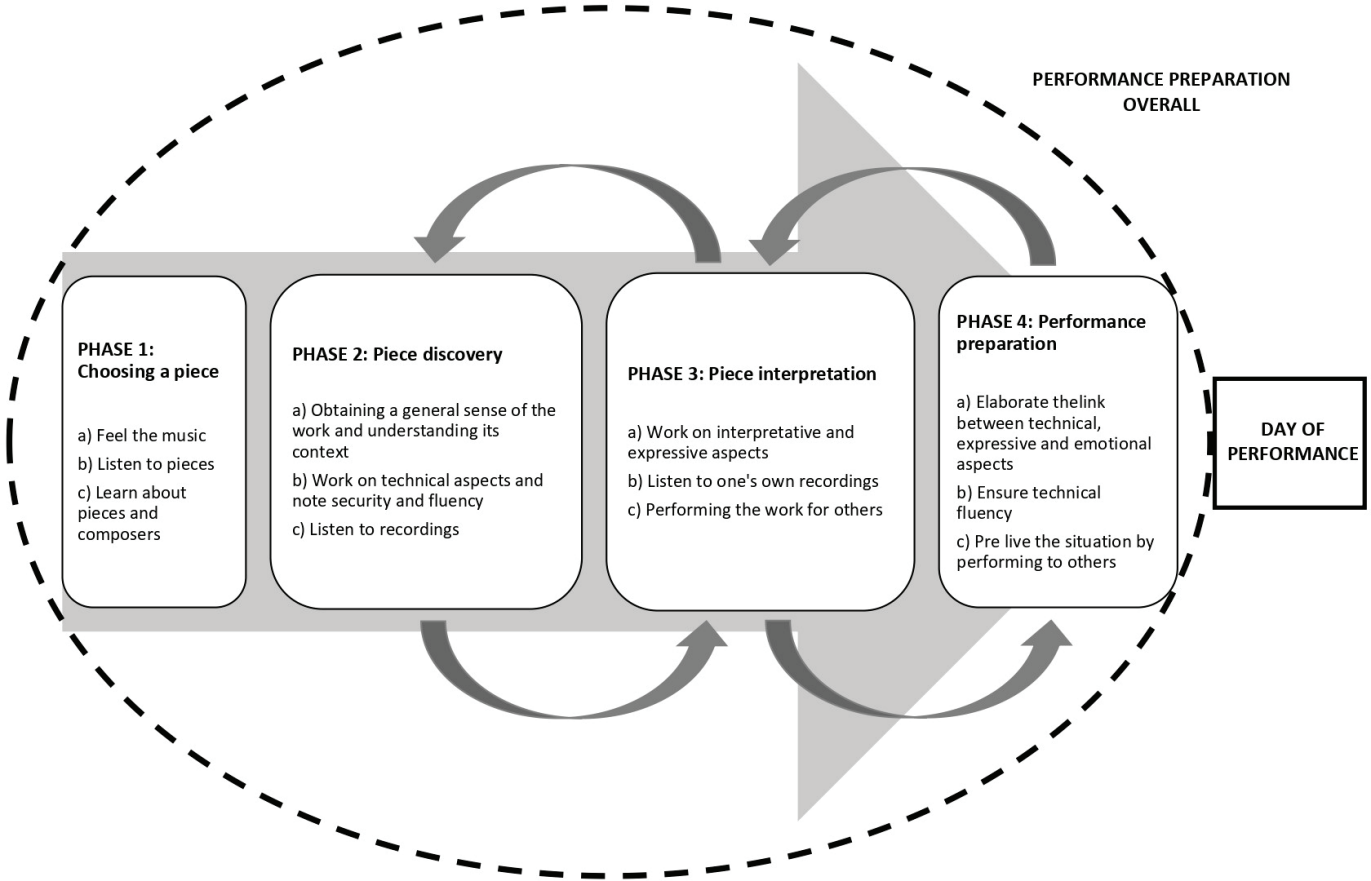
Participants’ phases, themes, and subthemes of a performance exam preparation.

Based upon the analysis, different themes emerged and categories were created. These were clustered into four overarching phases including Phase 1: Choosing a piece; Phase 2: Piece discovery; Phase 3: Piece interpretation, and Phase 4: Performance preparation, all of which formed the performance preparation overall (see Figure 2). In the subsequent sections, themes are discussed *via* their specific subthemes. These phases are interrelated to each other and conservatory musicians moved from one to the other depending on their needs. Emotions were particularly discussed by participants as they were intensely experienced; therefore they are presented as a transversal theme to the four phases. Participants reported that the duration of their preparation process was influenced or governed by their university’s annual academic calendar. Nonetheless, the total length of time that participants reported having to prepare for a performance ranged from 5 to 9 months.

### Phase 1: Choosing Pieces

Participants reported that the first task, when preparing for a performance, was to select the repertoire. This phase was composed of three subthemes lasting from 2 weeks to 1 month: (1) feel the music; (2) listen to pieces; and (3) learn about pieces and composers.

#### Feel the Music

When choosing repertoire, the students tended to focus on pieces that would be appropriate for their stage of development and therefore suitably challenging. They also searched for enjoyable pieces to practice and perform. Further to this point, some students spoke about choosing repertoire that they felt they had an emotional connection to and would also enjoy playing.

“I find it hard to work on pieces you don’t like at all during the three months. If I like my pieces it’s easier to work on them.” (M2).

“I choose the pieces I’m connected to. If I find a piece I love I would want to practice it a lot.” (M12).

#### Listen to Pieces

Listening to music appeared to be an important part of the musician’s preparation. Also, the music students tended to choose pieces that would allow them to demonstrate their musical skills and abilities.

“[I listen] to the repertoire [of] different musicians. Everyone plays styles differently and you have to find the best version of how it should be played.” (M2).

“I like pieces I like to listen to. And also, pieces that I know I’m going to be able to play. So not too hard and not too easy.” (M5).

#### Learn About Pieces and Composers

To be able to choose a piece, conservatory musicians seek an understanding of the context in which the music was created. The historical context and its technical particularities were considered important criteria when choosing and then committing to a piece.

“I really like reading about the story of the music and the composer and how the time was at the moment of the creation of the piece. I find this gives me more ideas for when I perform the piece.” (M1).

“I’ll be listening to recordings and doing the research around the pieces. Pieces have contexts, some pieces where written in the 1800’s and some had a tape going with it so it’s really new to play. It turned out that the piece had a rich context because it was written on the first synthesizer.” (M3).

“When I play 20th century pieces I try to look at the historical background to see what the composer went through.” (M6).

### Phase 2: Piece Discovery

After choosing a piece, the musicians reported that a form of “piece discovery” was the next step in the process. This phase was often composed of three subthemes lasting about 3–5 months: (1) obtaining a general sense of the work and understanding its context; (2) work on technical aspects and note security and fluency; and (3) listen to recordings.

#### Obtaining a General Sense of the Work and Understanding Its Context

Once a piece is selected, participants would set about trying to achieve a general sense of their chosen repertoire. Some participants acknowledged that this phase was hard work, but enjoyable and exciting.

“Yes it’s like each piece reminds you something and link it in an emotional way. If I think a piece is sad how am I going to play to convey this feeling to the audience. Sometimes I make up a story. [...] You get a sense of it in the first period, but it’s more in the second period.” (M3).

“It’s quite exciting because you find a piece and then you try to find where you can get to with the piece.” (M4).

#### Work on Technical Aspects and Note Security and Fluency

Participants in this phase would focus on getting the piece up to the required tempo and speed. Conservatory musicians reported focusing on getting the notes under their fingers and singers getting an understanding of the text by playing on the piano to learn the notes and pitches.

“If it’s a piece with a lot of fast technical passages, I’m going to practice technical exercises that work on my fingers for the sake of improving my technique and not just work on the piece.” (M3).

“Probably at the beginning it would be to memorize words. I’m going to play to memorize the entire piece and then do the tempo, the speed.” (M13).

#### Listen to Recordings

Listening in this phase was different than in Phase 1. Participants suggested that they needed to be more concentrated when listening, in order to understand how to convey the emotional side of the piece in their playing and combine this with their technique.

“I listen to recordings of the piece by different musicians. Everyone has different styles and you have to find the best version of how it should be played.” (M1).

“This mental gymnastic between technique and emotion is what takes place for around all the semester. So, it’s not just listening. I try to understand how I can combine technique and emotion. [...] So it’s being able to emotionally connect what it has to be with my fingers.” (M12).

### Phase 3: Piece Interpretation

In Phase 3, which lasted 1–2 months, participants recounted that determining and settling on an interpretation was a challenging process. Conservatory musicians entered this demanding phase of their performance preparation to get closer to what they wanted to present on the day of their performance. This phase was composed of three subthemes: (1) work on interpretative and expressive aspects; (2) listen to one’s own recordings; and (3) performing the work for others.

#### Work on Interpretative and Expressive Aspects

Having developed a general sense of the piece during Phase 2, music students reported turning their attention to interpretive and expressive aspects within Phase 3. Although participants still refined technical aspects of their repertoire, often this was undertaken alongside developing their confidence, finesse, and trust in their repertoire’s knowledge. Increasing familiarity with their pieces was reported to enhance comfort and confidence.

“Now it’s about starting to develop an emotional understanding of the piece and what is important to me to try and convey to the audience, what I want to bring as a performer to the piece, what is different.” (M4).

“The aim of [Phase 3] is about finesse, making sure that I am capable, that I don’t panic because it can happen. I develop trust in my instrument which are my ears and my voice. It is more about trusting that I am able to do what I need to do.” (M13).

#### Listen to One’s Own Recordings

The importance of listening to one’s own audio recordings appears to be linked to this third phase where some of the conservatory musicians employed different strategies (e.g., mirror, postponed listening). This appeared to allow a better appropriation of the music, technically, but also emotionally.

“And I started to record myself. I put my camera down and I looked at my performance. [I look at] my facial expression. Because when I play something I have a bad facial expression.” (M8).

“Yeah it’s also good to record yourself playing with the accompanist. And check like the dynamics and if you didn’t go soft at this point or another. Look at the music and evaluate yourself.” (M9).

“What I would do is I would record when I was at the piano and then straight after I listen to it and I continue make practice and I keep the recordings. And then, the next day, I do it again maybe even a week later, I listen to it because sometimes you get a different perspective when you come back.” (M13).

#### Performing the Work for Others

By this stage, music students were starting to shift their focus to performing their pieces in front of others. This involved running through their pieces, rehearsing with accompanists, and performing for others. As such, some participants actively sought out opportunities to practice performing for others (e.g., friends, family, peers) as part of their larger performance preparation.

“In class we have weekly classes in front of [teacher and friends] where we can play or you can sign up on Wednesday there’s moment where you can play and teacher gives you feedback.” (M2).

“You can also perform in one of the classes every week in front of students and it’s recorded [...] and [the teacher] will give you feedback.” (M9).

### Phase 4: Performance Preparation

Conservatory musicians reported that the last task was the preparation for a performance exam. They spoke of working to develop and attain a certain performance level and mindset. This phase was composed of three subthemes lasting from 2 weeks to 1 month: (1) elaborate the link between technical, expressive, and emotional aspects; (2) ensure technical fluency; and (3) pre-live the situation by performing for others.

#### Elaborate the Link Between Technical, Expressive, and Emotional Aspects

With this aim, the conservatory musicians spoke of ensuring that the technical and emotional aspects of their pieces were well connected. Expressive aspects of the piece were focused on during this phase.

“I mean it’s easier to be dramatic when you know the piece really well. You can feel it and project it to the audience. So, doing work on the dramatic side of it in the last period. You can focus on it more at the end.” (M9).

“Then it’s focusing on actually getting expression from the pieces and try to put a personal touch to it. [...] In that period it’s developing expression and how you want to present it. It’s polishing what I have decided.” (M13).

#### Ensure Technical Fluency

With the aim of getting their pieces performance ready, the overwhelming majority of the practice behaviors reported by the participants were directed toward ensuring they had attained a certain level of technical control over their repertoire.

“It’s playing musically and making sure you have the technique. And make sure every phrase is musical.” (M2).

“Once I’ve identified the parts I’m not happy with, I try to figure out if it was a performance-related issue or a mechanical issue. If I can play it correctly in the practice room I make sure I pay attention while performing, and if it’s a mechanical issue I practice until it works.” (M5).

#### Pre-Live the Situation by Performing for Others

Conservatory musicians spoke about performing for others and how this helped them pre-experience the situation, develop confidence for playing in the “real” performance, and enhance their stage presence. The participants also reported employing a range of strategies to help them prepare for the specific conditions experienced within their performances.

“Ideally at this stage I would like to not be worried about whether I can play or not, but whether I can play well. And I also need to feel that I can communicate in a large room where people are listening. I’d say this is key to performing, and that I know exactly what it is that I want to say.” (M7).

“I feel more mentally at ease when playing the program if I’ve already visualized myself doing it”. (M10).

“My main objective is to put myself into stressful situation in order to be able to play at the recital. For example, I try to play my pieces first thing in the morning without having the time to practice them before.” (M11).

### Emotional Responses Throughout Phases 1–4

Emotional responses through all phases varied considerably. From enjoyable and exciting to stressful and overwhelming, conservatory musicians faced multiple emotions they had to manage and deal with. However, some specificities can be pointed out for each phase.

The first phase seems to be enjoyable, but some music students also felt this period to be quite stressful, because it was the beginning of their preparation and they could see all the work they had to provide.

“I find this phase quite stressful because you want to get things ready as soon as possible but it’s complicated to process everything. In the first month it feels like it’s never going to end. I feel like ‘oh my god I still have so much to do!’” (M2).

The second phase seems the hardest in terms of commitment. Hard work is required, but it is also an enjoyable and gratifying phase because music students see the progress they make.

“I like the second phase the most because it feels like you’ve progressed and you’ve developed but it doesn’t matter if you make mistakes. You’re still learning at this point. It’s a more gratifying period. You know what you want to do and you progress every day.” (M2).

Also, due to the length of this phase, it was reported by some of the conservatory musicians that they experienced boredom and found the learning process monotonous.

“[Phase 2] gets monotonous. You have to spend a lot of time on a single piece. You sometimes get bored but I try to play other pieces not to be bored.” (M6).

However, increasing familiarity with their pieces was reported to enhance comfort and confidence.

Perhaps, due to the proximity of the upcoming performance, many participants reported negative emotional responses in Phase 3 which manifested as apprehension, stress, and worry due to the performance’s proximity:

“I think Phase 3 is the most difficult. I’m getting close to the recital. I get a little nervous and some days I feel like I’m not ready and I worry that I will fail.” (M1).

For some participants, this apprehension and worry seemed related to concerns about the outcome or mark they might receive for their performance exam. Also, related to concern about evaluative aspects of their upcoming performances, it seems emotional responses depended upon how prepared conservatory musicians felt they were:

“Giving performances can be a wonderful experience, if you know your pieces. But if you don’t know your pieces it can be terrible!” (M7).

Unsurprisingly, nerves and performance anxiety were reported to be a concern for music students, particularly in the two last phases. In an attempt to manage nerves, participants discussed employing strategies to overcome their anxiety, including ensuring a sufficient level of preparation before the performance.

## Discussion

This study sought to explore the context-specific experiences and self-regulation efforts that music students live during their preparation period, leading up to an important performance.

Previous studies have highlighted findings that musicians who have accumulated more hours of deliberate practice reach a higher level than those who practice less (Lehmann and Ericsson, 1997; Jørgensen, 2002). However, the quality of practice can also determine the level of achievement (Williamon and Valentine, 2000; McPherson, 2005). Quality is not quantity, but it seems that musicians prioritize quantity over quality (Pecen et al., 2018). However, what does quality practice refer to in the music domain? As discussed by various authors (Biasutti and Concina, 2014; Pecen et al., 2018), music students in our research pointed out the importance of being well prepared and developing strategies to manage and overcome performance nerves and factors such as stress and anxiety, that are associated with their performance. However, it seems that many also lacked tools and strategies to overcome these challenges. Our results highlight different phases participants go through; however, what we found also is that the students do not appear to plan this preparation upstream of the learning phases. It seems that this preparation tends to emerge unconsciously. These findings are consistent with existing research in music performance reporting a lack or absence of planning and goal-setting behaviors (e.g., Talbot-Honeck, 1994; Hatfield, 2016). Also, our results present varying opinions on playing in front of others. Indeed, some conservatory musicians seek situations during which they can play in public and others avoid playing in front of others. However, even if they seek feedback from others, they often delay the time of confrontation as late as possible. A fairly marked predominance seems to concern the tendency to focus on technique and musicality rather than the specifics of the preparation phases. Moreover, conservatory musicians’ calendar within a music university system is often tight and full, therefore it appears difficult for them to plan and pace the preparation needed to master the repertoire they have to perform for the exams. Self-regulation learning (SRL) seems to be important to overcome challenges musicians face during the preparation and learning process and ensure a certain quality of practice (Zimmerman, 1998; McPherson and Zimmerman, 2011; McPherson et al., 2019). Miksza (2015) showed that musicians who received self-regulation instructions were able to make significantly greater gains in performance achievement than those who were simply presented with instruction about practice behaviors (e.g., repetition, memorization). Essentially, SRL involves cyclical and multi-layered processes comprising three complementary phases: planning, doing, and reflecting (Leon-Guerrero, 2008; Upitis et al., 2010). Planning strategies and organizing time seem to positively influence musicians’ ability to confront and overcome a performance (e.g., stress) (Araújo, 2016; McPherson et al., 2019). However, our results do not highlight these planning competencies. Indeed, musicians do not seem to plan their preparation period.

In our results, conservatory musicians reported varying experiences and time spent in each phase (i.e., from 2 weeks to 5 months). In other domains, such as sport, preparation phases are structured according to very specific needs and the time variable is considered before the beginning of the preparation phase (Blumenstein and Orbach, 2015; Fletcher and Sarkar, 2016). A key skill of SRL is to be able to reflect, evaluate, and re-organize a plan previously designed (McPherson et al., 2019). In our research, conservatory musicians did not seem to re-organize their plan. Instead, they went through different phases quite similarly without questioning their effectiveness: (1) Choose, (2) Learn, (3) Perfect, and (4) Consolidate. Organizing a plan could be proposed to these conservatory musicians in order for them to be more able to prepare for their future performances. Also, the time spent in each phase varied significantly and a lot of time was spent in learning (Phase 2) and perfecting (Phase 3) their pieces for the performance exam. Far less time was given to challenging themselves in front of others (Phase 4). Some attempts were undertaken to ask peers and family about their performance, regardless of their expertise, but these were most often sought in the last part of the preparation period. On the one side, a lot of time was spent perfecting pieces, technically and musically (i.e., 4–7 months), and on the other side, less time was used to consider feedback from others before a performance exam (i.e., 2 weeks to 1 month). In this way, these results are consistent with Stamer’s (2004) results.

Therefore, and as discussed by Pecen et al. (2018), conservatory musicians need guidance on how to optimize their preparation process. Challenging oneself by presenting their work to others appears to be crucial for learning and perfecting expertise. Our findings show that this presenting to others appears only in the last phase. Regarding the preparation tools, it appears that students report making greater use of recordings as their expertise develops (Hallam et al., 2012). However, recordings or performing for others could be used sooner in order to optimize the time the students have to prepare for a performance exam. In addition, conservatory musicians are alone in their process of performance preparation, especially in the last phase, with only rare support from peers, teachers, and their close environment. Therefore, an increasing number of music schools offer orchestra audition simulations that help musicians prepare for demanding performance situations (Williamon et al., 2014). The main aim of these simulations is to help music students acquire a better idea of what the performance will feel like and be able to cope with any distracting factors underlying performance, such as stage fright, stress, and, in general, performance-related emotions (Aufegger et al., 2017).

Additionally, emotional regulation is a key aspect of SRL (Clark et al., 2014; Thomson and Jaque, 2017) and to regulate emotionally people use coping strategies (Lazarus and Folkman, 1984). It seems that organizing, disorganizing, and re-organizing their preparation throughout the period could help them cope with emotions and negative affect (Thomson and Jaque, 2017). Developing conservatory musicians’ competencies in self-regulated learning would help them cope with stress and negative effects encountered by the preparation of a performance exam. In our results, conservatory musicians did not seem to use specific strategies to deal with emotional challenges encountered (e.g., stress). They faced the emotions and affects induced by a specific phase without specific strategies to change or increase related positive emotions. Conservatory musicians could develop flexible tactics to appropriately respond to and improve an emotional affect induced by a specific context (e.g., monotony, anxiety) (Thomson and Jaque, 2017).

Finally, according to our findings and, despite the number of successful intervention studies in the literature, practical advice on self-regulated learning in music performance enhancement processes is somewhat sparse. Initiatives are developing globally (e.g., Chesky et al., 2006; Liertz and Macedon, 2007; Williamon et al., 2014), yet even more proactive and integrated approaches are called for by researchers and students alike (e.g., Parncutt, 2007; Weller, 2008, 2010; McPherson et al., 2019). Furthermore, although a growing body of research demonstrates the benefits of performance psychology in music (e.g., Osborne, 2013; Osborne et al., 2014; Braden et al., 2015), institutions appear to be resistant toward changes. Academic work is often reduced to allow as much time as possible for practice (Parncutt and Williamon, 2005; Weller, 2010) as performers often consider everything that is not practice “a waste of time” (Brown, 2012).

There are a number of limitations of this qualitative study. The interviewer was experienced in performing elicitation interviews with musicians. However, as she is not an expert musician, she was sometimes unfamiliar with expressions commonly used in the music community. Thus, during the interview, she asked conservatory musicians to explain their expressions when needed, to ensure the accuracy of the information collected. Also, only 13 music students participated in the study. Therefore, it is not possible to generalize these results. Furthermore, the participation in this study was voluntary; therefore, there is no homogeneity in the group of students. We did not test the students’ level of practice; however, they are all seeking to work and specialize in the music field. It would be interesting to ask professional musicians about their performance preparation. And, it is important to note that all participants in this study were Australian, leading to a culture-specific context constraining the generalization to other cultures. The musical educational system might be different depending on the country. Finally, self-preservation and retrospective recall bias have also to be considered, even so this bias was compensated through a written graph of their preparation process (Pecen et al., 2018).

## Conclusion and Practical Implications

In conclusion, the aim of this study was to explore the context-specific preparation of conservatory musicians and highlight the self-regulation efforts they develop to get ready for a performance exam. Different working models were underlined: some conservatory musicians develop and employ very specific and planned preparation programs, whereas others just play and work on everything at the same time. The educational system does not explain how to develop a performance exam preparation process. This study suggests that it is important for young musicians to learn how to master a performance exam, not by proposing a plan, but by instructing and accompanying them in the development of self-regulation strategies. Music students should be supported in how to organize, disorganize, and plan their preparation process. A guide could be developed based upon evidence-based practice that details how music students can prepare for an exam or an important deadline and the resources they can use to overcome associated challenges (Leon-Guerrero, 2008). Also, besides music students, teachers and university music schools should be qualified and skilled to relay their competencies to young musicians and students (McPherson et al., 2016, 2017). Given the apparent relevance of self-beliefs and self-regulated learning to musicians’ success, it would be useful for researchers to explore conservatory musicians’ perceptions of themselves and their skills, as well as how those perceptions influence practice behaviors and performance experiences (Leon-Guerrero, 2008).

Consequently, initiatives, if available, can often be mostly theoretical and lacking in practicality and systematic delivery (e.g., lecture or workshop formats are typical) (Weller, 2008). Although clearly well intentioned, such theoretical formats appear to lack traction with student-performers (Brown, 2012) and do not accurately reflect performance psychology training. Indeed, structured, systematic, and comprehensively interdisciplinary training that practically communicates the tools necessary to optimize exam preparation for musicians is uncommon. An approach that might encourage more autonomy and engagement would be to ask students to use a practice journal to record and reflect on the strategies they choose in order to maintain effort, to monitor accuracy, correct errors, and develop their own interpretation of the piece. Such efforts to develop greater engagement have been found by McPherson et al. (2012) to increase practice efficiency. In addition, accompanying music students in the development of self-regulated skills to prepare for exams or recitals could help them experience these events in a more serene way and thus improve their general well-being and health (Williamon and Thompson, 2006; Kreutz et al., 2009; Antonini Philippe et al., 2019).

## Data Availability Statement

The datasets generated for this study are available on request to the corresponding author.

## Ethics Statement

This study involving human participants was reviewed and approved by Human Research Ethics from the University of Melbourne. The participants provided their written informed consent to participate in this study. Written and informed consent was obtained from all participants attesting that the data could be unrestrictedly analyzed and discussed for publishing.

## Author Contributions

RA and GM contributed to the conception and design of the study. RA, CK, NV, and TC organized the database. TC, RA, CK, and NV performed the analysis. All authors co-wrote the manuscript, contributed to the manuscript revision, read, and approved the submitted version.

### Conflict of Interest

The authors declare that the research was conducted in the absence of any commercial or financial relationships that could be construed as a potential conflict of interest.

## Nomenclature

M1: Musician 1
M2: Musician 2; etc.
